# Electric Scooter Trauma in Rome: A Three-Year Analysis from a Tertiary Care Hospital

**DOI:** 10.3390/jcm14103615

**Published:** 2025-05-21

**Authors:** Bruno Cirillo, Mariarita Tarallo, Giulia Duranti, Paolo Sapienza, Pierfranco Maria Cicerchia, Luigi Simonelli, Roberto Cirocchi, Matteo Matteucci, Andrea Mingoli, Gioia Brachini

**Affiliations:** 1 Department of Surgery, Sapienza University of Rome, 00185 Rome, Italy; bruno.cirillo@uniroma1.it (B.C.); mariarita.tarallo@uniroma1.it (M.T.); giuliaduranti95@gmail.com (G.D.); paolo.sapienza@uniroma1.it (P.S.); luigi.simonelli@policlinicoumberto1.it (L.S.); andrea.mingoli@uniroma1.it (A.M.); gioia.brachini@uniroma1.it (G.B.); 2 Department of Emergency Surgery, Policlinico Umberto I, 00161 Rome, Italy; 3 Department of Medicine and Surgery, S. Maria Hospital, University of Perugia, 06123 Terni, Italy; roberto.cirocchi@unipg.it; 4 Department of Surgery, Università degli Studi di Milano, 20122 Milano, Italy; matteo.matteucci@unimi.it

**Keywords:** electric scooters, trauma, road injury, injury prevention, helmet use

## Abstract

**Background:** Electric motorized rental scooters (ES) were introduced in Italy in 2019 as an alternative form of urban transportation, aiming to reduce traffic congestion and air pollution. As their popularity has grown, a parallel increase in ES-related injuries has been observed. This study aims to investigate the types and patterns of ES-related injuries and to identify potentially modifiable risk factors. **Methods:** We conducted a retrospective analysis of all consecutive patients admitted to the Emergency Department of Policlinico Umberto I in Rome between January 2020 and December 2022 following ES-related trauma. Collected data included demographics, injury mechanisms and types, helmet use, Injury Severity Score (ISS), blood alcohol levels, and patient outcomes. **Results:** A total of 411 individuals presented to the Emergency Department due to ES-related injuries, either as riders or pedestrians. The mean age was 31 years (range: 2–93); 38 patients (9%) were under 18 years of age. Fifty-six accidents (14%) occurred during work-related commutes. Only three riders (0.7%) wore helmets, and nine patients (2%) had blood alcohol levels > 0.50 g/L. Cranial injuries (134 cases, 32%) and upper limb fractures (93 cases, 23%) were the most frequently reported serious injuries. The mean ISS was 4.5; 17 patients (4%) had an ISS ≥ 16. A total of 270 orthopedic injuries and 118 (29%) maxillofacial injuries were documented. Head trauma was reported in 115 patients (28%), with 19 cases classified as severe traumatic brain injuries. Twenty-three patients (5.5%) were hospitalized, three (0.7%) required intensive care, and one patient (0.2%) died. **Conclusions:** ES-related injuries are becoming increasingly common and present a significant public health concern. A nationwide effort is warranted to improve rider safety through mandatory helmet use, protective equipment, alcohol consumption control, and stricter enforcement of speed regulations.

## 1. Introduction

Electric scooters (E-scooters) have rapidly gained popularity worldwide as a convenient, low-cost, and environmentally friendly mode of transportation. Their widespread adoption has been driven by technological innovation and growing concerns over air pollution and urban traffic congestion. In Italy, E-scooters were first introduced in 2019 during the COVID-19 pandemic as an alternative to crowded public transportation. Their use has since remained widespread, supported by increased public awareness of air quality and the need to reduce emissions from conventional vehicles.

Air pollution is a major global health concern and has been identified by the World Health Organization (WHO) as a key contributor to all-cause mortality and to specific diseases, including chronic obstructive pulmonary disease, lung cancer, pneumonia, stroke, and ischemic heart disease. Motor vehicle emissions are among the most significant sources of ambient air pollution.

Despite their environmental advantages, E-scooters pose serious safety concerns. Road traffic injuries remain a leading cause of death among young people in the European Union, with 19,800 fatalities reported in 2021. Several studies have reported increasing numbers of E-scooter-related injuries and emergency department visits across Europe [1,2,3,4], the United States [5,6,7], and other parts of the world [8,9].

In Italy, the widespread availability of E-scooters through app-based rental services has contributed to their popularity in urban areas. Riders often appreciate their speed, accessibility, and ease of use. However, the number of injuries involving E-scooters has risen sharply. In 2021, road injuries in Italy increased by 28% compared to 2020, despite the presence of pandemic-related restrictions. According to the Italian National Institute of Statistics (ISTAT), there were 2101 reported E-scooter-related road accidents involving injuries or deaths in 2021, compared to 564 in 2020. These incidents resulted in nine fatalities—including one pedestrian—and 1980 injuries among riders and passengers.

Although E-scooters were introduced as a tool for sustainable urban mobility [10], the lack of comprehensive safety regulations [11], such as dedicated lanes or mandatory helmet use for all riders, has contributed to a growing number of accidents. E-scooters can reach speeds of up to 20 km/h on roads and 6 km/h in pedestrian zones, yet Italian law requires helmet use only for riders aged under 18. A driver’s license is not required to operate an E-scooter.

Despite the increasing incidence of E-scooter-related trauma, the scientific literature describing injury patterns and accident dynamics remains limited [12]. As the use of shared micromobility services continues to expand, research is urgently needed to guide preventive strategies and to improve clinical outcomes after trauma. Understanding the mechanisms, risk factors, and consequences of E-scooter injuries can inform both public health policy and trauma system preparedness.

The aim of this study is to analyze the characteristics and injury patterns associated with E-scooter accidents presenting to a large tertiary care hospital in Rome over a three-year period, and to identify modifiable risk factors to support the development of effective prevention and safety strategies.

## 2. Materials and Methods

### 2.1. Study Design and Setting

We conducted a retrospective observational study based on anonymized data from 411 patients consecutively admitted to the Emergency Department (ED) of Policlinico Umberto I, a tertiary care university hospital and level I trauma center in Rome, Italy. The study period spanned from January 2020 to December 2022. An electric scooter (ES)-related injury was defined as any traumatic event involving an electric rental scooter, either as a rider or as a pedestrian impacted by one.

### 2.2. Inclusion and Exclusion Criteria

All patients presenting to the ED for injuries directly or indirectly caused by an ES were included, regardless of age, sex, or injury severity. Exclusion criteria were incomplete documentation or injuries not clearly attributable to ES incidents.

### 2.3. Data Collection and Variables

Data were extracted from the hospital’s electronic health records and supplemented by paper documentation when necessary. The following variables were recorded:

- Demographics: age, sex, and occupation;
- Injury characteristics: anatomical location, mechanism (fall, collision with another vehicle, collision with a stationary object), and time of injury;
- Clinical information: mode of ED arrival (ambulance vs. walk-in), triage classification (based on the 5-level national urgency scale), helmet use, alcohol intoxication (blood alcohol level > 0.5 g/L), and drug use (if reported);
- Imaging: performance of X-rays or CT scans, including cranial CTs;
- Injury severity: Injury Severity Score (ISS), derived from the Abbreviated Injury Scale (AIS), calculated for each patient. Traumatic brain injury (TBI) was defined as any acute intracranial lesion confirmed on CT imaging;
- Outcomes: hospital admission, ICU admission, emergency surgery, discharge status, mortality, and length of stay (LOS).

### 2.4. Injury Severity Score (ISS)

The ISS is an anatomical scoring system that quantifies overall trauma severity. Each injury is assigned an AIS score (range 1–6) and mapped to one of six body regions. The highest AIS scores from the three most severely injured regions are squared and summed to obtain the ISS, which ranges from 1 to 75. A score of 75 is automatically assigned if any injury is classified as AIS 6 (unsurvivable). Higher ISS correlates with increased risk of mortality, complications, and prolonged hospitalization.

### 2.5. Statistical Analysis

All data were analyzed using R Studio, version 2023.09.1+494 (Posit Software, Boston, MA, USA), running R, version 4.3.2 (R Foundation for Statistical Computing, Vienna, Austria). Normality of continuous variables was assessed using the Shapiro–Wilk test. Normally distributed variables are presented as mean ± standard deviation (SD), while non-normally distributed variables are summarized as median and interquartile range (IQR). Categorical variables are reported as counts and percentages. Differences between groups were assessed using the Chi-square test for categorical variables and the Wilcoxon rank-sum test for continuous variables. Additionally, multivariable logistic regression was performed to identify independent predictors of hospitalization, TBI, and ISS ≥ 16. A *p*-value < 0.05 was considered statistically significant.

### 2.6. Ethical Considerations

This study was conducted in accordance with the principles of the Declaration of Helsinki. Due to its retrospective and anonymized design, approval from the Institutional Review Board was waived. Written informed consent for clinical treatment and scientific use of data was obtained from all patients at the time of hospital admission.

##  3. Results

### 3.1. Demographics and Accident Characteristics

During the three-year study period, a total of 411 patients presented to the Emergency Department (ED) with injuries related to electric scooter (ES) use, either as riders or as pedestrians struck by riders. The median age was 31 years (range: 2–93), with a mean ± SD of 31 ± 12.5 years. The age distribution is detailed in Table 1. Notably, 38 patients (9%) were younger than 18 years. The majority of patients were male (281; 68%). Pedestrians represented 1% of cases (4 individuals).

Fifty-six injuries (14%) occurred during work-related commutes. The temporal distribution revealed that 216 incidents (52%) occurred between Monday and Thursday, suggesting a potential association with routine commuting.

Regarding arrival modality, 52% of patients self-presented to the ED, while 40% arrived by ambulance. Triage severity was distributed as follows: 36 patients (9%) were assigned Code 1 (life-threatening), 87 (21%) Code 2 (urgent), 194 (47%) Code 3 (stable), and 94 (23%) Code 4 (minor). No cases were classified as Code 5 (non-urgent).

### 3.2. Mechanism of Injury and Severity

The most frequent mechanism of injury was falling without collision, observed in 336 cases (82%). Collisions with other vehicles occurred in 55 patients (13%), collisions with stationary objects in 3 patients (0.7%), and other mechanisms (including being struck or unexpected maneuvers) in 17 cases (4.3%).

Injury severity differed according to mechanism. Among the 55 patients involved in collisions, 42 (72%) arrived by ambulance, and 25.9% were triaged as Code 1. Their mean Injury Severity Score (ISS) was 4.7. In contrast, among those who fell without collision, 65% self-presented, only 5.7% were triaged as Code 1, and their mean ISS was slightly lower at 4.5. 

Helmet use was documented in only 3 patients (0.7%), and 9 patients (2%) had blood alcohol concentrations exceeding 0.50 g/L at the time of the incident.

### 3.3. Injury Distribution and Imaging

A total of 270 orthopedic injuries (66%) were recorded. Among these, upper limb fractures were observed in 93 patients (34%), while lower limb fractures occurred in 22 patients (8%). Maxillofacial injuries were identified in 118 cases (29%), and head trauma was reported in 115 patients (28%). Of the patients with head trauma, 19 (17%) were diagnosed with severe traumatic brain injury (TBI), including 9 cases of intracranial hemorrhage (2%) and 10 cases of cranial fracture (2%). Only 3 of the 115 patients with TBI were wearing a helmet at the time of injury.

More detailed breakdown of maxillofacial injuries revealed 20 nasal fractures (5%), 18 maxillary fractures (4.5%), 16 dental injuries (4%), 10 mandibular fractures (2.4%), and 5 zygomatic complex fractures (1.2%). Chest contusions were observed in 9 patients (2%), rib fractures in 16 (4%), pneumothorax in 5 (1%), and spine fractures in 6 patients (1.5%).

Regarding diagnostic imaging, 325 patients (79%) underwent X-rays, while 157 patients (38.1%) received CT scans. Of these, 108 (26.2%) were cranial CTs (Table 2).

### 3.4. Surgical Interventions and Hospitalization

A total of 24 patients (5.8%) underwent emergency surgical intervention. Notably, one case involved a vesical perforation caused by impact with the handlebars, which was managed successfully with laparoscopic bladder repair.

Hospital admission was required for 23 patients (5.6%), and 3 (0.7%) required admission to the intensive care unit (ICU). One death (0.2%) was recorded during the study period.

### 3.5. Injury Severity and Length of Stay

The mean ISS for the entire cohort was 4.5. Seventeen patients (4%) had an ISS ≥16, indicating major trauma.

The length of hospital stay (LOS) was significantly associated with head trauma severity. Patients with major TBI had a mean LOS of 5 ± 7.8 days, compared to 1.6 ± 3.1 days in patients with minor head injuries (*p* < 0.001). Among patients with major TBI, 52.9% required hospitalization for more than one day, compared to only 7.4% among those with minor TBI (Table 3 and Table 4).

### 3.6. Analytical Findings

Multivariable logistic regression analysis identified head trauma (OR 3.2; 95% CI: 1.4–7.6; *p* = 0.006), age over 40 (OR 2.1; 95% CI: 1.1–3.9; *p* = 0.02), and alcohol intoxication (OR 4.8; 95% CI: 1.6–14.4; *p* = 0.005) as independent predictors of hospitalization. Helmet use was inversely associated with the risk of severe traumatic brain injury, although statistical significance was not reached due to the low number of helmeted patients (OR 0.3; 95% CI: 0.06–1.2; *p* = 0.08) (Table 5).

## 4. Discussion

This study highlights the rapid rise and clinical impact of electric scooter (ES)-related trauma in an urban European context [13,14,15,16,17]. In our cohort, the majority of injured individuals were young adults aged between 20 and 39 years (64%), confirming prior evidence that road traffic injuries disproportionately affect active and working-age populations [18]. Interestingly, while previous studies have reported a higher incidence of ES-related trauma during weekends [19,20,21], our data showed a predominance of injuries during weekdays. This suggests increased ES use for commuting, particularly during high-traffic hours, and underscores the evolving dynamics of urban mobility.

Understanding the dynamics of E-scooter injuries is fundamental not only for prevention but also for anticipating complications and tailoring post-trauma care. Falls without collision were the most common mechanism of injury (82%), likely due to surface irregularities, lack of infrastructure, and rider inexperience. These injuries, although involving no external impact, often resulted in high-energy trauma due to sudden loss of balance or forward momentum—findings that are consistent with other reports [22,23,24]. Collisions, although less frequent (13%), were associated with higher injury severity, as reflected by elevated ISS scores and greater ambulance transport and triage urgency.

Consistent with the existing literature [25], concussions and orthopedic injuries were associated with increased likelihood of hospitalization. Regression analysis in our cohort confirmed that head trauma, alcohol intoxication, and age over 40 were independent predictors of hospital admission. Importantly, we identified a strong association between head trauma severity and length of hospital stay (LOS). Patients with major traumatic brain injury (TBI) experienced significantly longer LOS than those with minor head injuries (mean 5 ± 7.8 vs. 1.6 ± 3.1 days; *p* < 0.001), indicating greater resource utilization and prolonged recovery [26].

The economic impact of ES injuries should not be underestimated. A study from Barcelona estimated that e-scooter-related work accidents resulted in an average of 110 days off work and over EUR 1 million in insurance costs over two years [2]. Similarly, a trauma center in London reported a mean hospital cost of GBP 1482.46 per patient [27,28]. Although we did not collect direct cost data, these findings suggest that the financial burden of ES trauma is substantial and reinforces the urgency of preventive action.

From a biomechanical standpoint, ES-related trauma shares characteristics with blunt injuries seen in falls, cycling crashes, and pedestrian impacts. The absence of structural protection, combined with the standing posture of riders, predisposes riders to upper body and craniofacial injuries. In our study, head trauma and upper limb fractures were the most frequent injuries, reflecting typical impact patterns in unprotected riders.

Helmet use remains a crucial, yet controversial, preventive measure. Despite solid evidence supporting the efficacy of helmets in reducing the severity of head trauma [29], only 0.7% of our patients were wearing one at the time of injury. Italian law currently mandates helmet use only for individuals under 18 years old. However, nearly 30% of our cohort sustained TBI, and intracranial hemorrhage was documented in nine patients. Even though three helmeted riders still experienced TBI, the protective trend (OR 0.3; *p* = 0.08) suggests that helmet use likely mitigates the severity of head trauma. These findings support legislative reform to mandate helmet use for all ES riders, regardless of age.

Stricker et al. [30,31] observed a significant increase in mortality and neurologic complications following the repeal of a universal helmet law in Michigan. Our results align with those findings and reinforce the necessity of broader preventive measures. These should include mandatory helmet use for all riders, the creation of dedicated electric scooter lanes, strict enforcement of speed regulations, the promotion of high-visibility clothing for nighttime riding, and systematic monitoring of alcohol and drug use in urban mobility contexts.

In addition to cranial injuries, we observed a high incidence of extremity trauma. Upper limb fractures were particularly common and often occurred as riders attempted to break their falls. While these injuries are rarely fatal, they frequently require surgical intervention and may lead to long-term functional impairment. The literature suggests that the use of wrist guards and limb protectors, although uncommon, may reduce the incidence and severity of such injuries [32].

Pedestrians were also affected, albeit infrequently, underscoring the broader public safety implications of unregulated ES use. Accidents involving pedestrians highlight the need for stricter enforcement of shared space policies and urban design that supports safe coexistence among different transport modalities.

Finally, national traffic data confirm that road accidents did not decrease during the study period. Instead, ES trauma has added to the overall injury burden. With approximately 12,000 traffic accidents per year in Rome, the 411 ES-related cases recorded at a single center over three years represent a substantial and growing public health issue.

Taken together, our findings illustrate the urgent need for a comprehensive national strategy to regulate electric scooter use. Legislative changes, urban infrastructure improvements, and targeted educational campaigns are essential to reduce accident rates and their personal, medical, and societal impact.

This study has several limitations. First, it is retrospective and single-center in nature, which may limit generalizability. Data completeness relied on existing medical records, and underreporting of minor injuries or misclassification of ES involvement cannot be excluded. Furthermore, no formal power analysis was performed due to the observational design. The absence of follow-up data precludes conclusions on long-term functional outcomes or delayed complications. Nevertheless, the large sample size and the fact that Policlinico Umberto I is Rome’s primary trauma center confer a high level of representativeness and relevance for urban European contexts.

## 5. Conclusions

This study provides a comprehensive overview of electric scooter (ES)-related trauma in a large urban setting in Italy, with a focus on injury mechanisms, clinical outcomes, and modifiable risk factors. As the use of ES continues to expand across European cities, associated injuries have become a frequent and pressing challenge for emergency and trauma services.

Despite their reputation as a convenient and environmentally sustainable means of transportation, ES are associated with a significant risk of trauma, particularly among young adults. Our findings highlight that the absence of protective equipment—especially helmets—and the lack of dedicated infrastructure are key contributors to both the frequency and severity of injuries.

The majority of affected patients were healthy, working-age individuals who sustained preventable injuries, often with long-term consequences. The most severe cases involved traumatic brain injury (TBI) and upper limb fractures, frequently requiring hospitalization or surgery. Notably, TBI and ICU admission were more frequent among those not wearing helmets, underlining the protective value of headgear.

While this study is limited by its retrospective design and single-center setting, the high case volume and the role of Policlinico Umberto I as Rome’s main trauma referral center provide valuable insights into the dynamics of ES-related injuries in dense urban environments.

Ultimately, these injuries are largely preventable. National and municipal authorities must implement evidence-based policies to reduce the burden of ES trauma. These include universal helmet mandates, improved road infrastructure, dedicated ES lanes, and public education campaigns focusing on visibility, rider behavior, and alcohol avoidance. A coordinated and multidisciplinary prevention strategy is essential to ensure safer integration of micromobility devices into urban transport systems.

## Figures and Tables

**Table 1:** Age group distribution.

Age Group (Years)	Number of Patients	Percentage
0–9	4	1%
10–19	61	15%
20–29	166	41%
30–39	95	23%
40–49	47	11%
50–59	25	6%
60–69	10	2.5%
70–79	2	0.5%
≥80	1	0.2%

**Table 2:** Injury pattern and severity score.

Injury Type	Number of Patients	Percentage
TBI	115	28%
ICH	9	2%
Skull Fracture	10	2%
Maxillary Fracture	18	4.5%
Dental Injury	16	4%
Nasal Fracture	20	5%
Chest Contusion	9	2%
Rib Fracture	16	4%
Pneumothorax	5	1%
Upper Limb Fracture	93	23%
Lower Limb Fracture	22	5.5%
Spine Fracture	6	1.5%

**Table 3:** Abbreviated injury scale by body region.

Body Region	Mean AIS Score
Head/Neck	1.78
Thorax	1.53
Abdomen	2.00
Extremity	1.53

**Table 4:** Length of stay and head trauma correlation.

	Minor Head Trauma	Major Head Trauma	*p*-Value
**Number of Patients**	394	17	
**LOS > 1 day (n, %)**	29 (7.4%)	9 (52.9%)	<0.001
**Mean LOS ± SD**	1.6 ± 3.1	5 ± 7.8	<0.001

**Table 5:** Multivariable logistic regression analysis of predictors of hospitalization.

Predictor	Odds Ratio (OR)	95% Confidence Interval	*p*-Value
Head trauma	3.2	1.4–7.6	0.006
Age > 40 years	2.1	1.1–3.9	0.02
Alcohol intoxication	4.8	1.6–14.4	0.005
Helmet use	0.3	0.06–1.2	0.08

## Data Availability

No new data were created or analyzed in this study.

## Abbreviations

AIS	abbreviated injury scale
ED	emergency department
ES	electric scooter
ICH	intracranial hemorrhage
ICU	intensive care unit
ISS	injury severity score
TBI	traumatic brain injury
